# Acoustic monitoring of coastal dolphins and their response to naval mine neutralization exercises

**DOI:** 10.1098/rsos.170558

**Published:** 2017-12-06

**Authors:** Marc O. Lammers, Marian Howe, Eden Zang, Megan McElligott, Amy Engelhaupt, Lisa Munger

**Affiliations:** 1 Oceanwide Science Institute, PO Box 61692, Honolulu, HI 96839, USA; 2 Hawaii Institute of Marine Biology, PO Box 1346, Kaneohe, HI 96744, USA; 3 Engelhaupt Consulting, 4173 Ewell Road, Virginia Beach, VA 23455, USA

**Keywords:** dolphin, passive acoustic monitoring, behavioural response, anthropogenic sound, navy

## Abstract

To investigate the potential impacts of naval mine neutralization exercises (MINEX) on odontocete cetaceans, a long-term passive acoustic monitoring study was conducted at a US Navy training range near Virginia Beach, USA. Bottom-moored acoustic recorders were deployed in 2012–2016 near the epicentre of MINEX training activity and were refurbished every 2–4 months. Recordings were analysed for the daily presence/absence of dolphins, and dolphin acoustic activity was quantified in detail for the hours and days before and after 31 MINEX training events. Dolphins occurred in the area year-round, but there was clear seasonal variability, with lower presence during winter months. Dolphins exhibited a behavioural response to underwater detonations. Dolphin acoustic activity near the training location was lower during the hours and days following detonations, suggesting that animals left the area and/or reduced their signalling. Concurrent acoustic monitoring farther away from the training area suggested that the radius of response was between 3 and 6 km. A generalized additive model indicated that the predictors that explained the greatest amount of deviance in the data were the day relative to the training event, the hour of the day and circumstances specific to each training event.

## 1. Introduction

The use of underwater explosives and other high-amplitude impulsive sound in industrial activities, hydrocarbon exploration and military training is becoming increasingly common in many marine habitats around the world, raising concerns about impacts on marine fauna. Marine mammals are particularly vulnerable to these types of sounds because of their dependence on hearing and acoustic signalling for sensing their environment, finding prey and communicating with conspecifics [1,2]. Documented impacts on marine mammals from explosions and other impulsive sounds include behavioural modifications [3–6], increased rates of entanglement [7], temporary shifts in hearing sensitivity [8,9], and mortality [10,11].

The naval forces of many nations conduct mine neutralization exercises in coastal waters as part of their regular training. These exercises typically involve detonating explosives in areas often co-habited by various species of marine mammals and, therefore, have the potential to disturb, injure or kill animals occurring there. In March 2011 three common dolphins (*Delphinus delphis*) were killed at the US Navy's Silver Strand Training Complex off San Diego, California during a training exercise using underwater explosives [11]. In this particular case, the animals entered the training area after initiation of a time-delayed detonation that could not be safely aborted. The US Navy is required to comply with US federal laws designed to protect marine species, including the Endangered Species Act and the Marine Mammal Protection Act. As part of the regulatory process, the Navy must monitor and report on certain activities that have the potential to injure or kill marine mammals, such as underwater detonations (UNDETs). One of the principal monitoring goals is to increase understanding of how marine mammals respond to sonar, UNDETs or other stimuli that result in the anticipated ‘take’ of individual animals, defined as hunting, harassing, capturing or killing a marine mammal [12].

To address this need, a long-term passive acoustic monitoring (PAM) study was conducted between 2012 and 2016 at the Virginia Capes (VACAPES) W-50 mine neutralization exercise (MINEX) training range near Virginia Beach, Virginia, to document the spatial and temporal occurrence of odontocete cetaceans and to document their behavioural responses to UNDETs. The most commonly seen odontocete species in this area is the bottlenose dolphin (*Tursiops truncatus*), followed by rare sightings of harbour porpoise (*Phocoena phocoena*) and common dolphins (*Delphinus delphis*) [13]. There are at least three Western Atlantic coastal bottlenose dolphin stocks that potentially occur in this area: the northern and southern migratory stocks and the northern North Carolina estuarine stock, with a combined estimated abundance of 21 500 animals [14]. Recent small-boat survey effort within the W-50 MINEX training area documented seasonal variation in local bottlenose dolphin densities, with the highest abundance estimated in autumn at *N* = 1277 and lowest in winter at *N* = 37 [13]. In combination with low re-sighting rates between years, these data suggest that dolphins are seasonal but not year-round residents of the area, and recent and previous evidence support a northward movement in warm months and southward movement in cold months [13–15].

The VACAPES training complex was established in 1977 and MINEX training occurs in the W-50 range year-round with between one and four training events taking place each month [16,17]. The exercises involve one or more detonations per day over one to three days, typically of charges ranging in weight between 2.3 and 9.1 kg. The objectives of this study were to: (i) determine the daily, monthly and seasonal occurrence of dolphins near the primary location of MINEX training, (ii) quantify the acoustic activity of dolphins in response to MINEX training events, (iii) establish the distance from the training area at which a response is observable, and (iv) identify the predictive factors associated with dolphin acoustic response to training events.

## 2. Material and methods

### 2.1. Data collection

Passive acoustic monitoring was accomplished using bottom-moored ecological acoustic recorders (EARs), which use hydrophones with sensitivity of −193.5 dB and a flat frequency response (±1.5 dB) from 1 Hz to 28 kHz [18]. EARs were programmed to sample at a rate of 50 kHz for 180 s (3 min) every 360 s (6 min) (i.e. a 50% duty cycle), providing an effective recording bandwidth of approximately 0–25 kHz. This bandwidth was sufficient to detect the whistles and the low-frequency end of echolocation clicks produced by bottlenose dolphins and other delphinid species (e.g. common dolphins) potentially occurring in the VACAPES area. Harbour porpoise signals are centred at 130 kHz [19], well above the effective recording frequency of EARs and, therefore, were not considered in this study.

**Figure 1.**
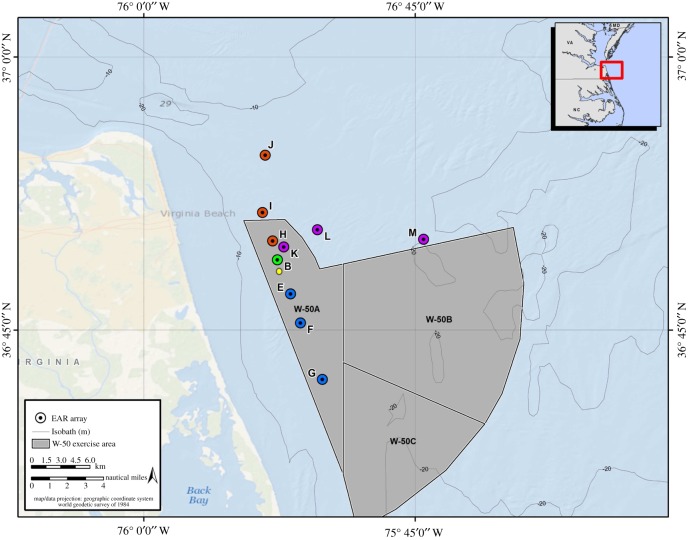
Spatial configuration of the three linear EAR arrays. Site B remained constant and north is shown in red (B–H–I–J), east in purple (B–K–L–M), and south in blue (B–E–F–G). The yellow dot represents the position of the ‘epicentre’.

EAR deployments took place between 15 August 2012 and 8 July 2016. The EARs were recovered, refurbished and re-deployed approximately every 2–4 months as weather conditions and access to the training range allowed. EAR clocks were set at the beginning of each deployment to local time (Eastern Standard Time or Eastern Daylight Savings Time). During the first four deployments, two EARs (A and B) were located 0.5–1 km apart, and their recording periods were offset so that one unit was recording while the other was off. As a result, one of the paired units was always ‘on’ in order to detect any nearby UNDETs. The two EARs were placed in 14 m water depth approximately 1 km to the north of a site that was identified as the ‘epicentre’ of MINEX training activity (figure 1). This is a search field location approximately 7 km offshore from Virginia Beach where the majority (approx. 95%) of MINEX detonations were expected to occur each year.

Beginning in September of 2013, two additional EARs were used in a modified deployment configuration. During alternating deployments, four EARs were placed in a ‘linear-array’ shape, which was aligned to the south, east or north. These EAR units were spaced at approximate distances of 1 km (site B), 3 km (sites E, H or K) 6 km (sites F, I or L), and 12 km (sites G, J or M) from the primary MINEX epicentre (figure 1). The 1 km EAR and 6 km EAR were programmed to record synchronously, and the 3 and 12 km EAR were programmed to record synchronously at an offset duty cycle from the 1 and 3 km EARs in order to ensure the capture of all UNDETS, as in the previous year. Site B was maintained as the 1-km location for all subsequent linear-array deployments to ensure the continuation of the data time-series from this site. The other deployment configuration in 2013–2016 consisted of site B and three other sites approximately 150 m from site B intended as a localization array; results from the localization effort are not presented here and only data from site B are included in the present analysis.

### 2.2. Data analysis

Dolphin occurrence at site B was analysed using data from August 2012 to August 2015 (table 1). An experienced acoustic analyst manually examined all available recordings obtained from site B for the presence/absence of dolphin signals during this 3-year period using the Matlab™ program Triton [20]. During deployment #7 (16 Feb–27 April 2014), the disk drive of EAR B malfunctioned so the data from EAR K (3 km from the epicentre) were analysed instead. Each 3 min recording containing dolphin signals (whistles, echolocation clicks or burst pulses) was considered a ‘detection’ of dolphins in the area, and dolphin occurrence was quantified in terms of the number of detections (i.e. number of recordings with dolphin signals present) per day. Because deployments made after August 2015 were intermittent and/or shorter compared to the first 3 years, they were not examined for dolphin occurrence. Three different analysts were employed sequentially over the course of the project and were trained for consistency using a common training dataset.

**Table 1.** EAR deployment/recovery information. Superscripts after EAR site indicate analyses conducted at that site: 1, UNDET detection; 2, dolphin presence/absence per recording; 3, dolphin acoustic activity during the days surrounding an UNDET (table 2).

EAR deployment	EAR configuration	begin recording	end recording	EAR sites	no. of explosions detected
1	paired EARs	15 Aug 2012	13 Oct 2012	A^1^ and B^1,2,3^	10
2	paired EARs	8 Dec 2012	25 Feb 2013	A^1^ and B^1,2,3^	3
3	paired EARs	16 Mar 2013	13 May 2013	A^1^ and B^1,2,3^	2
4	paired EARs	A: 1 June 2013B: 10 June 2013	A: 9 July 2013B: 22 Aug 2013	A^1^&B^1,2,3^	5
5	linear array (south)	21 Oct 2013	11 Nov 2013	B^1,2,3^,E^3^, F^a^ and G^3^	1
6	localization array (only site B incl. here)	16 Nov 2013	23 Jan 2014	B^1,2,3^	6
7	linear array (east)	17 Feb 2014	27 Apr 2014	B^a^,K^1,2,3^, L^3^ and M^3^	5
8	linear array (north)	19 May 2014	3 Aug 2014	B^1,2,3^,I^a^, H^3^ and J^3^	15
9	localization array (only site B incl. here)	16 Aug 2014	27 Oct 2014	B^1,2,3^	4
10	linear array (south)	10 Nov 2014	B: 23 Jan 2015E: 26 Dec 2014F: 17 Jan 2015G: 14 Nov 2014	B^1,2,3^,E^3^, F^3^ and G^3^	1
11	linear array (north)	9 Mar 2015	21 May 2015	B^1,2,3^, H^3^, I^3^ and J^3^	2
12	localization array (only site B incl. here)	25 June 2015	21 Aug 2015	B^1,2,3^	5
13	linear array (east)	13 Oct 2015	B: 15 Nov 2015L: 02 Dec 2015M: 5 Dec 2015	B^1,3^, K^a^, L^3^ and M^3^	1
14	linear array (south)	1 Feb 2016	23 Mar 2016	B^a^,E^3^, F^3^ and G^3^	6
15	localization array (only site B incl. here)	13 June 2016	8 July 2016	B^1,3^	2

^a^EAR lost or disk drive malfunctioned.

Analysts also manually searched for UNDETs in recordings. Beginning in 2015, an automated Matlab™ script was used to identify training events containing UNDETs by searching recordings for large deviations in acoustic amplitude from background levels with a near instantaneous rise time. Each UNDET was classified as ‘small’, ‘medium’ or ‘large’ based on the duration of the associated acoustic reverberations (0–2 s, 2–7 s and 7+ s, respectively), i.e. the period from initial onset to when acoustic energy fell below 6 dB above pre-UNDET ambient noise levels.

Dolphin acoustic activity was quantified in detail during timescales of seconds, hours and days surrounding UNDETs. For data from EAR B recorded during the first year of the project (Deployments 1–4), an analyst manually counted whistles in the 30 s before and after detected UNDETs; this analysis was not continued in subsequent years. In addition, all 3 min recordings on EAR B in the four days surrounding each MINEX training event were assigned a scalar acoustic activity index between 1 and 4 to represent the relative abundance of the various dolphin sounds detected, with greater values corresponding to greater signal abundance and variety of signal types (table 2). Recordings without dolphin signals were assigned a value of 0. The analysis period included the day before the event (to represent baseline activity levels), the day of the event, and the two days after each event. For training events occurring over multiple days, the day of the event was the day when the first UNDET was detected; the day after and the second day after were the two days following the final UNDET of the training event. Data from the three more distant EARs (3, 6 and 12 km) within the linear-array configuration in the latter 3 years of the project were also analysed (in addition to EAR B) for the day before, day of, and day after MINEX training events to investigate the acoustic behaviour of dolphins at greater distances from the training epicentre (table 1). Training events were excluded from analysis if they did not have a baseline period of 3 days prior to the event, in order to avoid confounding the ‘before’ and ‘after’ periods with other training events.

**Table 2.** Index values used to quantify dolphin acoustic activity in each 3 min recording made the day before, day of and the two days after each detected explosion, based on the abundance of dolphin whistles, burst pulses (BP) and echolocation clicks.

acoustic category	index value
no signals detected	0
1–20 whistles	1
BP only <10	1
echolocation only <2 clicks/s	1
21–40 whistles	1.5
echolocation only >2 clicks/s	1.5
BP only >10	1.5
echolocation and BP <10	1.5
1–20 whistles and echolocation or BP	2
> 41 whistles	2.5
echolocation and BP >10	2.5
1–20 whistles, echolocation and BP	3
21–40 whistles and echolocation or BP	3
21–40 whistles, echolocation and BP	3.5
> 41 whistles and echolocation or BP	3.5
> 41 whistles, echolocation and BP	4

Acoustic activity indices were used to quantitatively compare the acoustic behaviour of dolphins during the hours (site B) and days (all linear-array EARs) before and after MINEX training events throughout all years of the project. To determine whether the observed differences in the hours surrounding explosions were statistically significant, pairwise comparisons were conducted using a Wilcoxon signed-rank test between the hour before and the hour after, second hour after or third hour after an UNDET. Similarly, for the ‘day(s)’ time scale, hourly indices for each day before, day of, day after and second day after were averaged for the daytime hours (06.00–17.59) and night-time hours (18.00–05.59). The day before values were then matched with the corresponding day of, day after and second day after values and either a parametric paired *t*-test (for normally distributed data) [21] or a non-parametric Wilcoxon signed-rank test (for non-normally distributed data) [22] was performed. Seasonal variation in daylight hours was not explicitly accounted for in analyses; the ‘daytime’ hours do, however, represent the typical working day hours for naval exercises [17].

### 2.3. Predictive modelling

In order to test which predictors best explained the variation in dolphin acoustic activity in the hours and days surrounding MINEX training events, a generalized additive model (GAM) was constructed in the mixed GAM computational vehicle (mgcv) package using R software, version 3.3.1 [23–25]. The model was based on data recorded during deployments 1–14 (August 2012 to March 2016) by EAR B, 1 km away from the training epicentre. The response variable was dolphin acoustic activity, labelled ‘mean acoustic activity’ (MAA) in the dataset, which was quantified as the mean acoustic activity index for each hour of data recorded the day before, day of, day after or second day after an explosion event, normalized to be between 0 and 1.

MAA was tested as a function of nine predictors: season, hour of day, day/night, hours since explosion, size of explosion, number of previous explosions, day (relative to training event), deployment and cumulative hours (table 3). The first three predictor variables were chosen to account for the observed seasonal and diurnal variation in dolphin activity. The next four predictors were chosen because any changes in dolphin activity relative to MINEX training events were expected to be influenced by how close in time they were to explosions and the magnitude of the explosion or event. Deployment (number) was included to account for inter-deployment variation in conditions, and cumulative hours (elapsed since beginning of the dataset) was included to explain variation due to temporal autocorrelation.

**Table 3.** Predictor and response variables used in the model selection.

predictor	units	type	description	range/values
hours since explosion	hours	discrete, ordinal	counts the number of hours passed since the most recent explosion (not necessarily within same training event or deployment)	0–3169
hour of day	n.a.	discrete, ordinal	0 = midnight	0–23
size of explosion	n.a.	discrete, ordinal	0 = no explosion1 = small, 0–2 s long2 = medium, 2–7 s long3 = large, 7+ seconds longvalue is applied to each hour based on the size of the most recent explosion.	0–3
deployment	n.a.	categorical	no 1 km data for deployments 5, 7 or 14. Coded as fixed effect.	1, 2, 3, 4, 6, 8, 9, 10, 11, 12, 13
season	n.a.	categorical	autumn = Sept–Novwinter = Dec–Febspring = March–Maysummer = June–Aug	autumn, winter, spring, summer
previous explosions	n.a.	discrete, ordinal	cumulative count of the number of explosions that occurred within a training event	0–4
day	n.a.	categorical	defines when a recording occurred relative to an training event	day before, day of, day after, second day after
day/night	n.a.	categorical	based on sunrise/sunset times	day, night
cumulative hours	hours	discrete, ordinal	the number of hours that have passed since the beginning of the dataset (to account for temporal correlation). Coded as fixed effect	1–27 816
response	units	type	description	range/values
mean acoustic activity	n.a.	discrete, ordinal	average acoustic activity index for each hour of each day. Acoustic activity index ranged from 0 to 4 based on the number of whistles, burst pulses and echolocation present in the recording. Divided by 4 to fit a β-regression bounded by 0 and 1	0–1

A GAM with a *β* distribution and *logit* link was chosen for the starting model because the response variable (MAA) was bounded between zero and one. The residuals from the resulting model appeared to have fairly constant variance and showed only slight departures from the expected normal distribution. GAMs with *β* distributions were also fit to test the *probit*, *cloglog* and *cauchit* links. The different link functions did not have much influence on the residuals or explained deviance. Therefore, the *logit* link was used in further analysis.

The optimal combination of the nine predictors was determined with *dredge* in the *MuMIn* package [26]. All possible combinations of predictors generated a set of models ranked by corrected Akaike information criterion (AICc). The best candidate models (within two *Δ* AICc of the lowest AICc) all included hours since explosion, hour of day, deployment, season, cumulative hours and day as predictors (table 4). The model with the lowest AICc was selected as the ‘best-specified’ model as it was the simplest of the three.

**Table 4.** Top three models within two *Δ* AICc from output of dredge of the starting model.

model ID	predictors in model	*R*^2^	d.f.	AICc	*Δ* AICc
246	intercept + day + deployment + cumulative hours+ hour + hours since explosion + season	0.3359	36	−2733.3	0
502	intercept + day + deployment + cumulative hours+ hour + hours since explosion + season + size of explosion	0.3361	37	−2732.2	1.13
248	intercept + day + day or night + deployment + cumulative hours+ hour + hours since explosion + season	0.3361	37	−2731.8	1.52

To address autocorrelation, the predictor variable cumulative hours was included as described previously. Remaining dependence (autocorrelation) among residuals was tested using *acf* and the Durbin–Watson test in the *lmtest* library [27]. The *acf* function plots a correlogram of the residuals, displaying the approximate correlation at each time lag. The Durbin–Watson test checks for serial correlation between the residuals of the model. The *acf* plot suggested some autocorrelation exists; however the Durbin–Watson test indicated that autocorrelation was not a significant problem (*p *= 0.764).

All analyses were performed using R with the base, *dplyr*, *car*, *MuMIn*, *lmtest*, *mgcv* and effects packages [23,25–30].

## 3. Results

### 3.1. EAR deployments

Fifteen EAR deployment cycles were conducted during the study period (table 1). Two instruments were lost, but were replaced, and one instrument malfunctioned. In total, 609 584 3 min recordings were made among all EARs totalling 30 479 h of data. Of these, 213 176 recordings totalling 10 659 h of data were made at site B, representing coverage of approximately 888 days of the 1423 day study period.

### 3.2. Dolphin occurrence near ‘epicentre’ area of W-50

**Figure 2.**
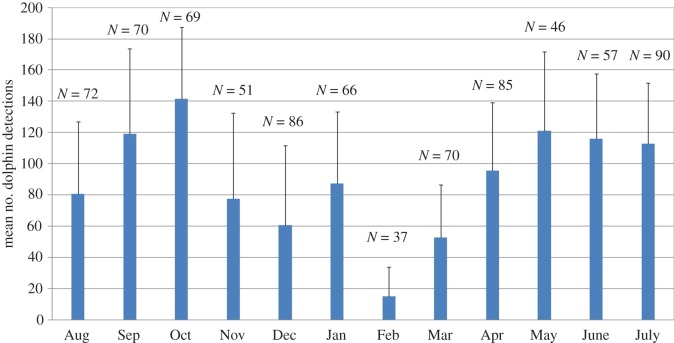
Number of daily dolphin detections at site B averaged by month for the 3 years of data collected between 15 August 2012 and 30 August 2015. Error bars represent one standard deviation. ‘*N*’ values give the total number of days that were monitored during each month.

Dolphin presence/absence at site B was analysed for the period from 15 August 2012 to 30 August 2015 (deployments 1–12), totalling 799 days of recordings. Dolphins were present almost daily from August 2012 to August 2015 in or near the MINEX range, with detections made on 97% of recording days. During the 3 year period analysed, a clear seasonal trend was observed in the mean number of daily detections each month. Dolphin detection rates (detections/day) were greatest between the months of April and October, decreased substantially between November and March, and were reduced by 90% during the month of February compared to the peak in October (figure 2). However, although the mean number of daily detections decreased during winter months, dolphins were still detected in the area nearly every day throughout the year.

### 3.3. Dolphin acoustic response at site B

**Figure 3.**
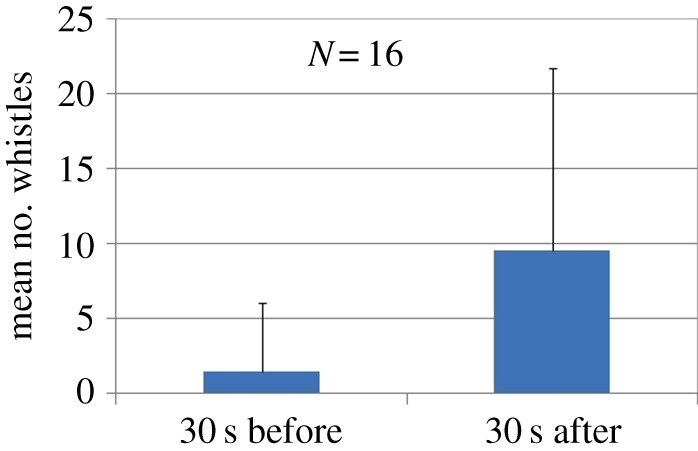
Number of whistles in the 30 s before and after UNDETs (*N* = 16) analysed in deployments 1–4 (Aug 2012–July 2013). Error bars represent standard deviation.

In total, 74 UNDETs were detected between 15 August 2012 and 8 July 2016, representing 38 MINEX training events. Of these, 16 UNDETs during 12 training days were analysed from site B during the first year of the project (August 2012–July 2013) to compare the number of whistles in the 30 s before and after an UNDET. The number of whistles increased significantly in the 30 s after explosions compared to the 30 s before (figure 3; Wilcoxon signed-rank test, *N* = 16, *Z* = 2.8, *p *< 0.005, effect size *r* = 0.50).

For the more comprehensive analyses at the hours and day scales, 31 of the 38 total training events coincided with data successfully obtained from site B, whereas seven training events took place during two deployments when the EAR at site B was either lost or malfunctioned. Of the training events recorded at site B, 31 included baseline data obtained from the day before the event, 30 from the day after the event, and 29 from the second day after the event. The disparity in the number of days recorded is due to the timing of EAR recovery and recording duration relative to two of the training events.

**Figure 4.**
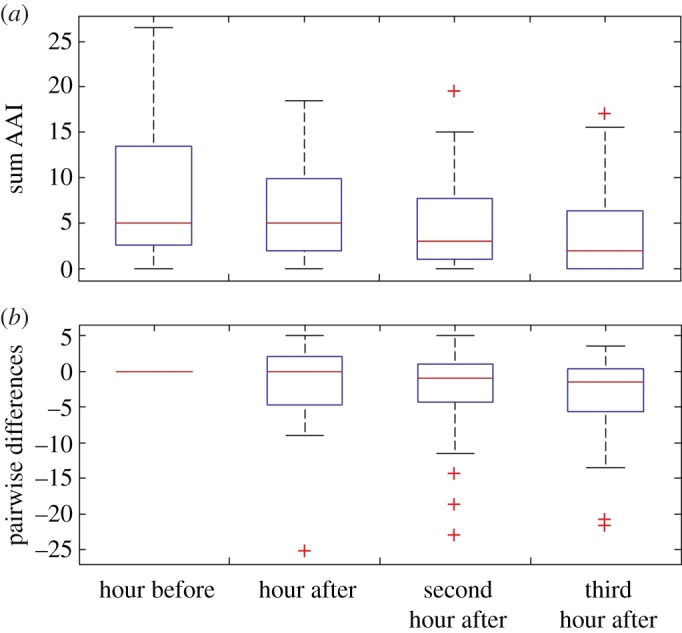
(*a*) Sum of acoustic activity index in the hour before, hour after, second hour after and third hour after explosions (*N* = 31). (*b*) Pairwise differences between the hour before and each of the 3 h after an explosion. Red lines represent median, box boundaries represent 25th and 75th percentiles.

Dolphin acoustic activity at site B decreased over the 3 h following an UNDET compared to the hour before (figure 4). This decrease was statistically significant for the second hour after and third hour after compared to the hour before (Wilcoxon signed-rank test, *N* = 31, *Z* = 2.29, *p *= 0.02, effect size *r* = 0.41, and *N* = 31, *Z* = 2.77 *p *= 0.006, *r* = 0.50, respectively).

**Figure 5.**
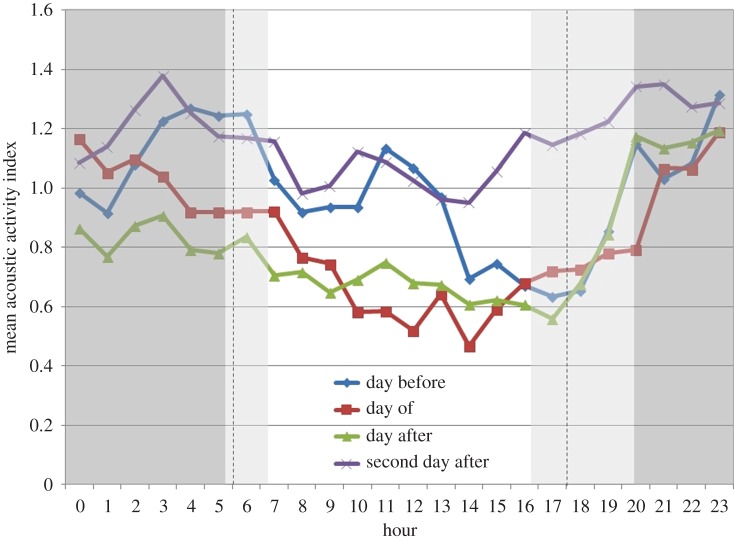
The hourly dolphin acoustic activity determined over the 24 h period of the day before (*N* = 31), the day of (*N* = 31), and the first (*N* = 30) and second (*N* = 29) day after MINEX training events at site B, all deployments. Dark shading represents night-time hours; light shading represents range of seasonal variation in sunrise and sunset times; dashed lines delineate the working hours used to group data by ‘day’ or ‘night’ [before, of or after].

Dolphin acoustic activity in the days surrounding a MINEX training event varied depending on the time of day. During the day before an event, dolphins were most active during midday (11.00–12.00 local time) and night-time hours (20.00–06.00 local time) (figure 5). On the day of MINEX training and the following day (day after), the midday activity peak was reduced or absent. The reduction in daytime dolphin acoustic activity on the day of and day after was significant compared to the day before an event (Wilcoxon signed-rank test, *N* = 31, *Z* = 3.46, *p *< 0.001, effect size *r* *=* 0.62 and *N* = 30, *Z* = 2.15, *p *= 0.032, *r* *=* 0.39, respectively). However, on the second day after a training event, daytime dolphin acoustic activity was significantly higher than the day before an event (Wilcoxon signed-rank test, *N* = 28, *Z* = 2.07, *p *= 0.038, effect size *r* *=* 0.39). By contrast, during night-time hours, no significant differences were found between dolphin acoustic activity levels from the day before compared to the day of (paired *t*-test, *t* = 1.092, d.f. = 30, *p *= 0.283, effect size Cohen's *d* = 0.20), day after (paired *t*-test, *t* = 0.692, d.f. = 30, *p *= 0.494, Cohen's *d* = 0.12) or second day after (paired *t*-test, *t* = 1.642, d.f. = 27, *p *= 0.112, Cohen's *d* = 0.31).

### 3.4. Dolphin acoustic response along linear array

**Figure 6.**
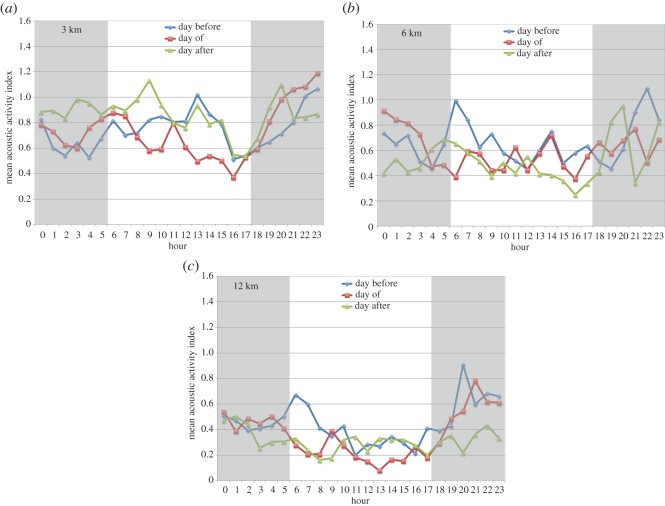
(*a–c*) The hourly dolphin acoustic activity determined over the 24 h period of the day before, the day of, and the day after MINEX training events detected during linear array deployments. Data are pooled for 3 km sites (*N* = 15), 6 km sites (*N* = 10) and 12 km sites (*N* = 14), regardless of directional orientation of array.

Seven linear-array deployments were conducted between September 2013 and March 2016. Overall, the baseline (day before) mean acoustic activity indices at the 3 km and 6 km sites were approximately 15% lower than EAR B, and the mean index at sites 12 km away was approximately 40% lower than EAR B (figure 6). However, hourly patterns in baseline dolphin acoustic activity at the linear-array sites were similar to site B, with elevated activity at night and a peak in activity during the daytime, although the magnitude of the daytime peak decreased with increasing distance from EAR B (figure 6). To statistically infer whether training events influenced dolphin acoustic activity at distances of 3, 6 and 12 km from the epicentre, the hourly indices for each day before, day of and day after were averaged for the daytime and night-time. Because more than 90% of recorded UNDETs occurred between 10.00 and 17.59, the hypothesis was tested that daytime effects would be observed between these hours at distances further away from the epicentre on the day of the training event. The night-time hours examined were those between 18.00 and 05.59, while the day after daytime hours considered were those between 06.00 and 17.59. The values corresponding to the equivalent day before periods were then matched with the corresponding day of and day after values and either a parametric paired *t*-test or a non-parametric Wilcoxon signed-rank test was performed.

Dolphin acoustic activity on the day before, day of, and day after training events varied depending on the distance of the monitoring site from the epicentre. For the pooled 3 km data (*N* = 15 MINEX training events recorded), the mean daytime dolphin acoustic activity on the day of the training event was significantly reduced compared to the day before (paired *t*-test, *t* = 2.26, d.f. = 14, *p *= 0.040, Cohen's *d* = 0.58). No significant differences were found between the daytime hours of the day before and the day after, or between the night-time hours of the day before and either the day of or day after. At 6 km from the epicentre (*N* = 10 MINEX training events recorded), no significant differences in dolphin acoustic activity were found between the day before and the day of or the day after for either the daytime or night-time periods. Lastly, for the pooled 12 km data (*N* = 14 MINEX training events recorded), the mean daytime dolphin acoustic activity on the day of the training event was significantly lower than the day before (Wilcoxon signed-rank test, *N* = 14, *Z* = 2.41, *p *= 0.016, effect size *r* = 0.64). However, at the 12 km sites, no significant differences were found between the daytime hours of the day before and the day after, or between the night-time hours of the day before and either the day of or day after.

**Figure 7.**
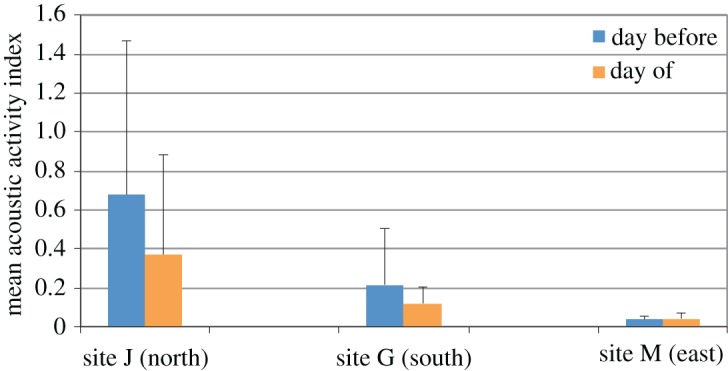
The mean acoustic activity index determined for sites J (*N* = 6), G (*N* = 5) and M (*N* = 3) during the day before and the day of a MINEX training event. Error bars represent one standard deviation.

To further explore the significant reduction in daytime acoustic activity at the 12 km sites during the day of the training event, data were grouped according to the linear array orientation (north, south or east). At the northern site, the baseline (day before) mean dolphin acoustic activity indices were more than three times greater than the southern site and more than 18 times the activity levels observed at the eastern site (figure 7). At both the northern and southern sites, mean daytime dolphin acoustic activity decreased by nearly 50% during the day of a training event. At the eastern site, activity levels remained low and unchanged.

### 3.5. Predictive model

The best-fitting model was determined to be the MAA as a function of day, deployment, cumulative hours, hour of day, hours since explosion and season. The explained deviance of this model was 39.07%. The deviance explained by each individual predictor was found by refitting the model without that predictor, then comparing the explained deviance in the new model to the explained deviance of the original model. Deployment, hour of day, and day (relative to a training event) were the predictors that explained the most deviance (table 5).

**Table 5.** Deviance explained by the whole model and each predictor.

predictor	deviance explained without predictor	difference in deviance explained from total
whole model	—	0.3907213
deployment	0.3455138	0.045208
hour	0.3473501	0.043371
day	0.3615639	0.029157
season	0.3746857	0.016036
hours since last explosion	0.3786422	0.012079
cumulative hours	0.3831953	0.007526

**Figure 8.**
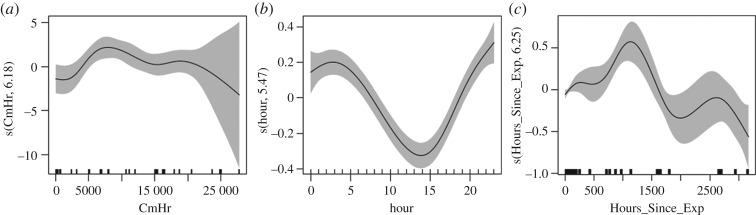
Smoothing splines from the best-fit model for each continuous variable: (*a*) cumulative hours ‘CmHr’, (*b*) hour of the day ‘hour’, and (*c*) hours since the previous explosion ‘Hours_Since_Exp’. The solid lines depict the marginal effect of the predictor while the other variables in the model are controlled. The shaded areas represent two standard errors. The *y*-axis indicates that the fit type is a thin plate regression spline and states the estimated degrees of freedom. The rugplot (short upward ticks along x-axis) indicates the location of the data samples (i.e. MINEX training events).

Figure 8 shows the smoothing splines from the best-fit model for each continuous variable. The standard error for the cumulative hours variable increases as the number of cumulative hours increases beyond approximately 20 000 h, probably due to the small number of data points late in the dataset (figure 8*a*). The cumulative hours plot indicates that MAA varies across the dataset, with lower values occurring in the first five months (0–3900 h) and final approximately 7 months of the dataset (greater than 22 000 h) and greater values during the 2 years in between 3900 and 22 000 h. MAA as a function of hour of day increased during the night-time hours of 19.00 to 08.00 and decreased during the daytime hours of 08.00–19.00 (figure 8*b*). From 60 h up to approximately 2 months after an explosion (approx. 1625 h), the number of hours since the previous explosion (hours since explosion) had a positive effect on MAA, while shorter and longer durations appeared to have a negative effect (figure 8*c*) (tables 4 and 5).

**Figure 9.**
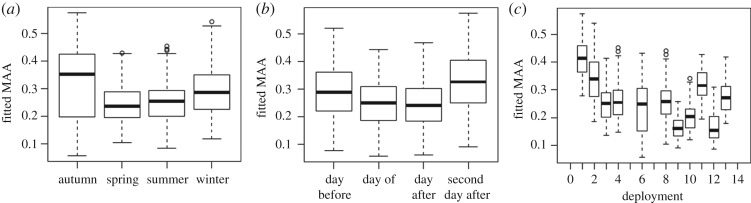
Predicted values of mean acoustic activity versus each categorical predictor: (*a*) season, (*b*) day relative to an explosion and (*c*) the deployment number.

Dolphin mean acoustic activity is plotted against categorical variables in figure 9. MAA was greatest in the autumn and lowest in the spring (figure 9*a*). MAA decreased on the day of and day after a MINEX training event, whereas on the second day after, MAA increased relative to baseline levels of the day before (figure 9*b*). There was also variation in acoustic activity as a function of deployment (number) (figure 9*c*), indicating that differences in deployment-specific conditions influenced MAA.

## 4. Discussion

Dolphins were detected nearly daily in the MINEX W-50 training area from August 2012–August 2015. Seasonally, there was a period of low occurrence or reduced acoustic activity during the winter months with a minimum in February. This finding is consistent with reported seasonal trends in bottlenose dolphin abundance offshore of Virginia Beach [13,15]; this is by far the most commonly sighted delphinid in this region [13] and the majority of detections in this study are presumed to be of this species. Other delphinid species such as common dolphins are sighted infrequently, but may also have been detected occasionally. The near-continuous presence of dolphins in the MINEX training area indicates that dolphins are exposed to noise from UNDETs, although it is not clear at what range most exposures occur.

There is strong evidence that dolphins respond behaviourally to MINEX training events. The data from site B, comprising 31 monitored training events in 4 years of data collection, paint a clear picture: after a short-term increase in whistling rates in the seconds following an UNDET (figure 3), dolphins either moved away or became less acoustically active during the hours following UNDETs (figures 4 and 5). Dolphin acoustic activity returned to baseline levels in the evening and night-time hours of that day (figure 5). However, during the daytime hours of the following day, acoustic activity was again reduced compared to baseline levels, but activity normalized again in the evening and at night. Interestingly, during daytime hours of the second day following a training event, dolphin acoustic activity at site B was generally higher than the baseline period.

It cannot be confirmed in the present study whether the observed changes represent individuals moving away from the area, a shift in acoustic signalling behaviour, or both. If the former is true, then the observed patterns suggest that dolphins temporarily move away from the epicentre during the day of the training event as well as the day afterward, but return during night-time hours (although it cannot be determined from this study whether the same individuals were recorded). Perhaps because training events often occur over multiple days, dolphins may anticipate additional UNDETs beyond the final day of the training event, which could explain the reduced acoustic activity observed during the day after the final UNDET. In other words, dolphins may hedge against potential future exposure to an UNDET by avoiding the area. The increase in dolphin acoustic activity on the second day following the training event may indicate that dolphins occupied the area in greater numbers, but this cannot be confirmed at present.

Alternatively, acoustic activity levels may also be a function of individual dolphin sound production rates. In a study of wild Western Atlantic coastal dolphin populations, vocalization rates varied geographically and as a function of behavioural state, and decreased with increasing group size at most locations [31]. The higher acoustic activity levels determined two days after a training event could indicate more frequent signalling by dolphins, perhaps reflecting altered behaviour and/or group sizes compared to the baseline. In captive animals, stressful events can lead to periods of reduced or no acoustic activity lasting hours or even days [32,33]. It is not known whether free-ranging animals respond similarly, but lower acoustic activity levels the day of and day following a training exercise may also signify a stress response.

The data obtained from the EARs 3, 6 and 12 km away from the epicentre further help inform our understanding of the response by dolphins to MINEX training events. There is evidence that dolphin acoustic activity is reduced 3 km away from the epicentre during the day of an UNDET, but not 6 km away, which suggests that the dolphins potentially respond to UNDETs at ranges up to 3–6 km (figure 6). These results are consistent with studies of captive bottlenose dolphins and a beluga whale (*Delphinapteras leucas*), which demonstrated disruption to trained behaviours in response to simulated explosions at ranges of 1.5 to 9.3 km depending on the size of the charge [4]. Of note, however, is that a significant reduction in acoustic activity on the day of an UNDET was also observed at the two 12 km sites towards the north and south of the epicentre (figure 7). This suggests that animals from these areas responded to relatively distant UNDETs or other aspects of training exercises. It is unclear whether this response indicates animal movement, behavioural changes or both. One possibility is that the animals may be moving away from more distant areas toward the epicentre to exploit prey fauna harmed or disoriented by the UNDET, during the night-time hours in between or after training days when acoustic activity was at normal levels. Another possibility is that habituation to UNDETs exists among animals typically occurring approximately 6 km away, but not among those substantially further away, which may be exposed to training events less frequently. More detailed studies are needed to better understand the response by these more distant animals to the north and south of the epicentre.

For the purposes of this study, it was not possible or feasible to empirically determine detection distances of dolphin signals or UNDETs, as the locations of sound sources were not available (or able to be localized). Janik demonstrated the range of detectability could be up to 20–25 km for dolphin whistles at maximum source level in Moray Firth, Scotland [34]. However, this was a relatively deep water habitat and these distances were estimated in low noise and Beaufort 0 sea state. Previous studies of bottlenose dolphins in more comparable high-noise, shallow, mud/sand bottom habitats in the eastern US and western Australia reported lower source levels of whistles and estimated the active space of dolphin whistles to range from less than 1 km to 2–6 km [35–37]. In 2014, work was conducted to measure received levels of UNDETs and derive a semi-empirical model for the propagation of underwater detonations in shallow water in/near the VACAPES W-50 range [38]. Sound exposure levels and peak pressure are calculated for 1, 3, 6 and 12 km for typical MINEX UNDET charge weights in table 6. Using the equations in [38], the sound exposure level for a receiver at 12 km away from a 20 lb charge would be 173 dB re 1 µPa^2^ s (table 6). Because of this and the offset duty cycles between neighbouring EARs, it is likely that EARs in the linear array configuration recorded independent groups of dolphins, and also that dolphins 12 km from an UNDET were still exposed to the sound.

**Table 6.** Sound exposure level (SEL) (integrated over time period containing 90% of waveform) calculated for UNDETs using Soloway & Dahl [38] equation.

range (km)	charge weight (lbs, C4, converted to TNT equiv. in kg)	SEL (dB re 1 µPa^2^ s^−1^)	peak pressure (Pa)
1	5	183	39 139
1	20	187	65 976
3	5	177	11 310
3	20	181	19 065
6	5	173	5168
6	20	177	8711
12	5	169	2361
12	20	173	3980

The modelling effort corroborated the empirically derived results and suggested some additional factors to consider. The explanatory power of the variables day and hour of day supports the finding that dolphin acoustic activity varied significantly on the days during and after an event compared to the day before, as well as the consistent diel pattern observed in dolphin acoustic activity. The inclusion of the deployment variable suggests that MAA was influenced by deployment- or event-specific conditions, such as the number of dolphins present prior to and during the training event, ambient noise conditions and the precise UNDET locations relative to the 1 km EAR site. Other predictors included in the best-fit model were season, hours since explosion and cumulative hours. The variation in MAA in response to season was consistent with the observed peak dolphin activity in autumn and lowest activity in spring. The increase in MAA with increasing hours since explosion for time periods of 60 h to two months may indicate that dolphins gradually returned to natural activity levels after a training event. The decrease in MAA with more than two months since the previous explosion does not lend itself to meaningful interpretation because conditions could have changed or training events may have taken place between deployments (typically approximately 2 months in duration). The variation in MAA observed over the course of the study (as a function of cumulative hours) may reflect the fact that three different analysts manually scanned different periods of recordings, which roughly corresponded to the different shape of the MAA function for 0–5 months, the middle 2 years and the final approximately 7–8 months. Although analysts were trained for consistency, some subjective variability in assigning acoustic indices may have persisted. However, because the response to training events was statistically analysed in a pairwise manner, any inter-analyst variability would not have affected the results.

The biological implications, if there are any, of the documented responses cannot be resolved by the present study. However, among the deleterious effects at the organismal level that might be expected are: chronically elevated stress hormone levels, acute or cumulative hearing damage/loss, reduced access to foraging habitat, and/or increased energy expenditures to avoid training areas [1,39–41]. For example, Rolland *et al*. [39] provide evidence that exposure to ship noise was responsible for increased levels of stress-related hormones in North Atlantic right whales (*Eubalaena glacialis*). Such effects may result in reduced fitness and possible declines in reproductive rates at the population level. Other possible impacts could include shifts in distribution and local reduction in abundance; such effects were demonstrated in bottlenose dolphins (*Tursiops* sp.) in Australia as a result of chronic disturbance from tourism vessels [40]. More detailed data on received sound characteristics are needed to interpret responses and link sound exposure to potential population-level effects [41].

Follow-on work would be useful to investigate the conservation implications and to help the Navy improve mitigation strategies. For example, future studies could investigate sound exposure levels as well as the types (physiological, behavioural, etc.) and magnitude of individual and group responses, potentially with the use of short-term tags, focal follow studies, unmanned aerial vehicles, hydrophone arrays and other means. In addition, analyses of the existing data could target calls from other marine mammals, including baleen whales and pinnipeds that occur in the area, to determine whether they also exhibit an acoustic response to MINEX training. Finally, long-term monitoring using passive acoustics, visual, and/or other means should be considered in order to detect any changes over time in dolphin occurrence and behaviour, and other environmental and anthropogenic factors should be integrated into cumulative impacts studies.

## Data accessibility

The processed datasets supporting this study are available from the Dryad Digital Repository: http://dx.doi.org/10.5061/dryad.1j05f [42].
